# Supplementary figures and images for: Aspergillus fumigatus ffmA Encodes a C2H2-Containing Transcriptional Regulator That Modulates Azole Resistance and Is Required for Normal Growth
Source: mSphere. 2022 Feb 9;7(1):e00938-21. doi: 10.1128/msphere.00938-21 (PMC8826999; doi:10.1128/msphere.00938-21)

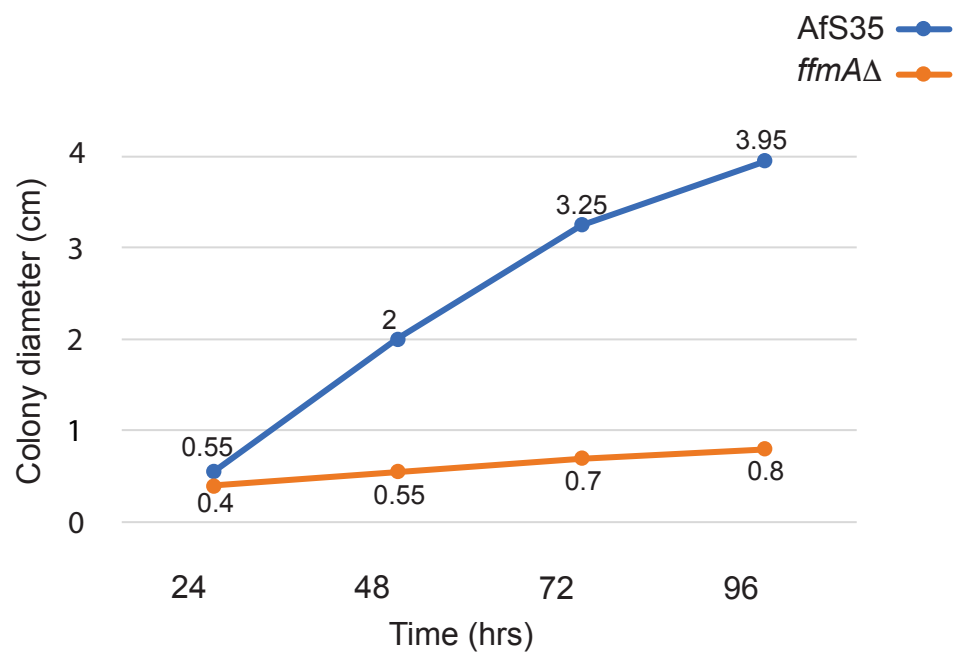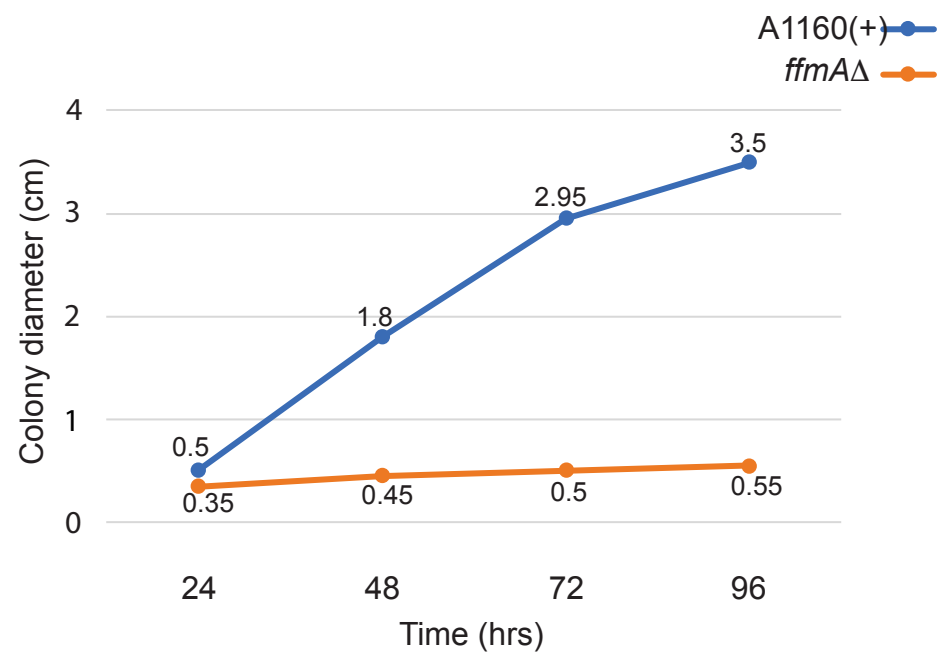

Supplement: FIG S1 [file msphere.00938-21-sf001.pdf]
